# Small Molecules against Metastatic Tumors: Concrete Perspectives and Shattered Dreams

**DOI:** 10.3390/cancers15164173

**Published:** 2023-08-18

**Authors:** Massimo Serra, Davide Rubes, Sergio Schinelli, Mayra Paolillo

**Affiliations:** Department of Drug Sciences, University of Pavia, Viale Taramelli 12, 27100 Pavia, Italy; davide.rubes01@universitadipavia.it (D.R.); sergio.schinelli@unipv.it (S.S.); mayra.paolillo@unipv.it (M.P.)

**Keywords:** metastasis, cancer, solid tumors, small molecules, tyrosine kinase inhibitors, cyclin dependent kinase inhibitors, KRAS inhibitors, integrin antagonists, RGD, anticancer therapy

## Abstract

**Simple Summary:**

Recent advances in anticancer drug research led to the approval of new small molecules with different mechanisms of action, therapeutic indications, and adverse reactions. In this review, small molecules recently approved for metastatic cancer therapy or in trials belonging to different pharmacological classes are described. Particularly, we focus on receptor tyrosine kinase inhibitors, which are the largest category, and on other small molecules interfering with cell cycle mechanisms. Special attention is devoted to mutated KRAS inhibitors, which probably represent the first dream come true in anti-metastatic cancer therapy of pharmacology researchers and open the way to potentially very broad therapeutic indications. A second section deals with another family of small molecules, integrin antagonists, that has gone through light and shade moments, and the molecules of still relevant interest are critically discussed.

**Abstract:**

Metastasis is the main cause of anti-cancer therapy failure, leading to unfavorable prognosis for patients. The true challenge to increase cancer patient life expectancy by making cancer a chronic disease with periodic but manageable relapses relies on the development of efficient therapeutic strategies specifically directed against key targets in the metastatic process. Traditional chemotherapy with classical alkylating agents, microtubule inhibitors, and antimetabolites has demonstrated its limited efficacy against metastatic cells due to their capacity to select chemo-resistant cell populations that undergo epithelial-to-mesenchymal transition (EMT), thus promoting the colonization of distant sites that, in turn, sustain the initial metastatic process. This scenario has prompted efforts aimed at discovering a wide variety of small molecules and biologics as potential anti-metastatic drugs directed against more specific targets known to be involved in the various stages of metastasis. In this short review, we give an overview of the most recent advances related to important families of antimetastatic small molecules: intracellular tyrosine kinase inhibitors, cyclin-dependent kinase inhibitors, KRAS inhibitors, and integrin antagonists. Although the majority of these small molecules are not yet approved and not available in the drug market, any information related to their stage of development could represent a precious and valuable tool to identify new targets in the endless fight against metastasis.

## 1. Introduction

Solid tumors are composed of different cancer cell types, generally considered to be derived from a single mutated cell clone that had undergone different mutations, according to their differentiation grade. Because the degree of differentiation of these cancers is directly correlated to their sensitivity to chemotherapeutic agents [1,2], the first cycles of chemotherapy are very effective in reducing the tumor mass. This therapeutic approach, however, has an important drawback whose effects become noticeable over time: the apoptotic cell death of cancer cells confers an advantage to less differentiated or undifferentiated cancer cells, which can grow in the absence of competition with other cancer cells for oxygen and nutrients. These less differentiated cancer cells, commonly referred to as cancer stem cells (CSC), can easily infiltrate healthy tissues and reach blood or lymphatic vessels, being responsible for resistance to chemotherapy and metastatic spread [3,4].

However, to colonize distant sites, these cells have to take a long and winding road that can be roughly divided into five steps: (1) expansion of original solid tumor mass; (2) proliferation and angiogenesis; (3) detachment and intravasation; tumor cells enter the circulation; (4) extravasation; circulating tumor cells settle at distant organ target sites; (5) local cell proliferation; change in the extracellular matrix (ECM) composition to support cell survival by shaping an appropriate tumor microenvironment. Any of the aforementioned steps implies the involvement of a plethora of different and very specialized components such as adhesion proteins, soluble factors, miRNAs, extracellular vesicles (EV), and immune system cells [5].

Quite surprisingly, despite several decades of intensive research devoted to unveiling the precise mechanisms of cancer cells spreading in the body, the involvement of key players in cell spreading is still poorly understood, and metastasis remains the primary cause of cancer mortality.

The (many) failures and (few) successes in the history of the search for effective and reliable antimetastatic drugs support the hypothesis that it is highly unlikely that only one molecule could exert an inhibitory effect, thus making the discovery of a “one-drug fits all” antimetastatic molecule a very daunting and elusive task.

Instead, the extreme complexity of molecular mechanisms involved in the metastatic process clearly reinforces the notion that only the combined effect of several small molecules with different molecular targets, maybe supported by other tailored pharmacological therapeutic strategies, might be effective to counteract the metastatic spread [6].

Classical candidate drugs for solid tumors with antiproliferative effects are mostly screened by their ability to induce tumor shrinkage, but unfortunately, this parameter does not correlate with cancer cell dissemination and, therefore, is not predictive of an antimetastatic effect.

The recent and spectacular rise of checkpoint inhibitors and monoclonal antibodies that block the activation of overexpressed membrane growth factor receptors changed the game in the treatment of solid tumors. However, the impact of checkpoint inhibitors on metastasis treatment, still in the early phase of clinical testing, is uncertain and controversial, thus requiring further validation and standardizations of methods dedicated to the assessment of their clinical efficacy [7].

Likewise, the limited efficacy of monoclonal antibodies in metastatic colorectal cancer, together with the finding that the pharmacological treatment with these drugs is not considered to be cost-effective, making their use as antimetastatic agents very problematic and uncertain in the future [8]. 

These considerations prompted projects in drug discovery to focus special attention on the wide variety of small molecules acting on different steps of the metastatic process [9,10,11,12].

In the pretentious attempt to partially help clinical oncologists and pharmacologists untangle this complex picture, in this paper, we describe some recent members of different classes of antimetastatic small molecules: intracellular tyrosine kinase inhibitors (TKIs); cyclin-dependent kinase inhibitors; KRAS inhibitors; and integrin antagonists.

We do believe that lessons learned from failures and successes gained from the study of these agents might give new insights into the molecular machinery of metastasis, thus improving the development of the next generation of reliable and effective antimetastatic agents [13].

## 2. Tyrosine Kinase Inhibitors

In recent years, the current widespread use in therapy, together with the growing number of new molecules in development, has clearly assessed the central and fundamental role of TKIs as valuable tools in the pharmacology of cancer.

This consideration is strongly supported by the large number of clinical studies currently ongoing. At the present time, the designing and screening of TKIs by drug companies is a topic field in metastatic oncology. A search in the portal https://clinicaltrials.gov, accessed on 20 June 2023, querying with the entries “metastatic cancers and TKIs” in the US retrieved 14 entries in phase I, 49 in phase II, 12 in phase III, and 1 in phase IV for clinical studies with reported results.

The common pharmacological action exerted by the majority of TKIs relies on the blocking of aberrant and dysregulated signaling pathways found in cancer cells [14]. For some selected TKIs, this blocking could be further dissected into two intracellular targets: (a) catalytic pocket of tyrosine kinase receptors (RTKs); and (b) different downstream kinases activated by the same or different RTKs [15]. Compared to other small molecules, TKIs offer some advantages, including the possibility of performing synthetic chemical modifications to optimize solubility and lipophilicity, the implementation of in vitro screening of lead compounds toward several oncogenic kinases, and last but not least, the advantage of the development of dedicated formulations for oral administration.

The issue of the ability of TKIs to diffuse into and be retained by distant and difficult-to-access compartments or tissues is very critical when dealing with the inhibition of metastatic processes. One example related to this problem deals with the clinical management of secondary brain metastasis treatment because drugs must cross the blood–brain barrier (BBB) to block prooncogenic kinases or aberrant signaling pathways found in metastatic cancer cells migrated into the brain.

Several recent strategies have been implemented to facilitate TKIs penetration into selected body districts [16]: (1) nanoparticle (NPs) formulations, such as polymeric NPs, gold NPs, or liposomes; (2) the concomitant administration of efflux transporter inhibitors (ETIs) to increase the bioavailability of drugs into the brain; and finally, (3) refinement of chemical design to enhance drug lipophilicity by in silico quantitative structure–activity relationship (QSAR) models.

Despite these limitations, small molecule inhibitors (SMIs) are one of the most effective and widely adopted classes of drugs in cancer therapy. In addition, notwithstanding the rushing impact of biotechnology drugs, Big Pharma’s investment in these molecules is constantly on a positive trend, with large numbers of approved new molecular entities [NME] each year [17].

The majority of approved SMIs have been employed in the pharmacological treatment of solid tumors, and only in recent years, new clinical studies describing the outcome of these molecules in metastatic cancer cells have been reported in the literature. The extreme complexity of players involved in the metastatic process, together with our still poor knowledge of the plethora of premetastatic endogenous kinases and dysregulated signaling pathways, make the identification of suitable targets a formidable challenge. Although these considerations suggest that the use of TKI drugs is still in its infancy, we anticipate that further clinical trials will expand their use in oncology. Here, we will briefly describe some of the features and outcomes of recently approved SMIs tested in clinical studies for their ability to counteract cancer cell spread in various solid metastatic tumors.

**Figure 1:**
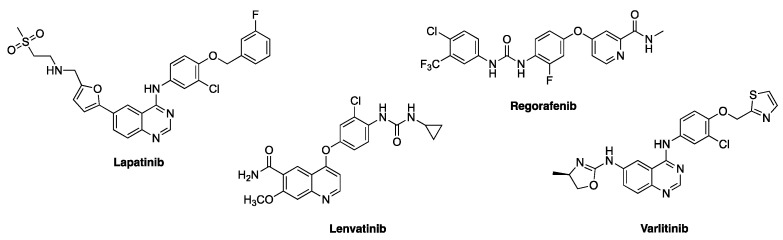
Selected tyrosine kinase inhibitors employed in anticancer therapy.

Lapatinib (Tykerb^®^, Tyverb^®^) (Figure 1) is an oral TKI whose mechanism of action includes blocking phosphorylation of the human epidermal growth factor receptor (EGFR or HER) 1, HER2, and HER4, extracellular signal-regulated kinase 1 and 2 (ERK-1, 2), and protein kinase B (PKB/AKT).

Lapatinib is currently employed in combination with capecitabine [18] to treat patients with advanced HER2-positive breast cancer following standard therapy with anthracyclines and taxanes or in combination with trastuzumab for patients with metastatic HER2-positive in prior chemotherapy [19]. Due to its lipophilic properties, lapatinib has been tested in animal models of brain metastasis. Indeed, in a mice model of brain metastases induced by injection of breast of HER2 cancer cell lines, selective accumulation of lapatinib in brain metastatic cells decreased HER2 phosphorylation and the extent of brain metastasis [16]. However, despite these encouraging results, brain bioavailability of lapatinib should be enhanced and improved because it is quickly extruded from the brain to blood via P-glycoprotein and breast cancer resistance protein (BCRP) transporters, thus limiting its efficacy in brain metastases. A review aimed at identifying prognostic factors and survival parameters after developing bone radioiodine-resistant metastases (BM), together with the effects of combined therapies in patients with HER2+ breast cancer (BC), reported that a combination of trastuzumab and lapatinib therapy or trastuzumab and pertuzumab therapy had the longest median survival compared with other therapies [20]. Quite notably, the efficacy of combined therapies using lapatinib could be extended to other tissues colonized by metastatic breast cancer cells because dual HER2 blockade with trastuzumab and lapatinib inhibited tumor growth in patient-derived xenografts of HER2-amplified metastatic colorectal cancer [21]. As a title of example, lapatinib is in a clinical trial for a number of solid tumors sharing the features of high EGFR and/or HER1/2 expression, particularly high-grade gliomas (NCT02101905), prostate cancer (NCT00246753), and head and neck cancers (NCT01044433).

Among approaches exploited to increase TKIs penetration of the BBB, nanoparticle (NPs) systems seem the most promising and performant; (a) NPs cross BBB easily and display enhanced permeability and retention in the tumor site; (b) NPs can be used as a shuttle system for many drugs and finally, (c) biodegradability of NPs limit their toxicity. In this context, a nanomedicine system co-loaded with lapatinib/doxorubicine and stabilized with glycol chitosan showed a potent therapeutic effect toward triple-negative breast cancer cells in comparison to a mixture of free drugs [22].

In cancer cells, characterized by extreme complexity and interactions among dysregulated convergent and divergent cell signaling transduction pathways, the resistance mechanism of redundancy may represent the main cause of the failure or lack of efficacy of the SMI treatment [23]. In redundancy, different elements of the signaling transduction pathways may act on different elements in the same biological or dynamic manner to converge on a common target, and therefore, the inhibition of one of these elements by a compensatory mechanism does not affect the whole biological outcome in terms of combined responses of the cell. Redundant elements compensate for the blocking of targeted genes by recruiting and leveraging the ability of crosstalk, among other pathways, to overcome the effects of SMI treatment in cancer cells. To circumvent this serious obstacle, the oncology community devoted considerable efforts to the chemical synthesis of SMIs that are less specific and with a broader range of inhibition toward driver kinases involved in redundancy [24]. Among these SMIs, varlitinib, regorafenib, and lenvatinib have recently shown promising results in clinical studies designed to test their efficacy in different types of metastatic cancers.

The idea that a non-specific TKI could give better therapeutic results compared to other selective compounds led to the design of varlitinib, a pan HER inhibitor originally thought for the treatment of metastatic cancers overexpressing HER receptors [25]. Varlitinib, also known as ASLAN001, is a potent small molecule targeting HER1, HER2, and HER4 that inhibits the phosphorylation of these receptors in vitro at concentrations (range 2-7 nM) comparable and even lower than lapatinib [26]. Since biliary tract cancers (BTC) have been found to overexpress HER receptors, a number of clinical studies were designed to test varlitinib in combination with other cancer therapies. These promising in vitro features, however, did not give the expected results because a recently terminated clinical trial (NCT03093870, 26) showed no significant improvement compared to capecitabine treatment alone. Other trials have demonstrated that varlitinib is well-tolerated (NCT03082053, NCT03368846), but therapeutic benefits for BTC have not been demonstrated at the moment. Nevertheless, the phase IIb trial for metastatic breast cancer (NCT02396108) is currently ongoing to confirm and extend promising results obtained with a 300 mg dose twice a day [27].

Regorafenib (BAY 73-4506, Stivarga^®^) is a novel oral multikinase inhibitor approved for the treatment of metastatic colon rectal cancer (CRC) and found to improve progression-free survival (PFS) in patients with metastatic gastrointestinal stromal tumors (GIST) [28]. Regorafenib, developed in a screening project aimed at enhancing the potency of sorafenib, differs from the parent drug in the presence of a fluorine atom onto the central aromatic ring (Figure 1). The main mechanism of action of regorafenib relies on the inhibition of various RTKs, including c-KIT, RET, BRAF, VEGFR1–3, TIE2, PDGFR-β, and fibroblast growth factor receptor (FGF-1). Other cellular effects of regorafenib include a decrease in tumor growth and lymph node metastasis, interrupted tumor cell–MSC interaction, and modified tumor-supporting stroma [27]. However, some reports have investigated the possibility that the real mechanism of regorafenib could be broader than previously thought, being not limited to the inhibition of TKIs. For example, regorafenib treatment was found to upregulate the level of PUMA, a p53 target, and a critical mediator of apoptosis, thus promoting apoptosis induction in different colorectal cancer cell lines [29]. In another study, regorafenib treatment enhanced the progression-free survival and metabolic responses via downregulation of the AKT/mTOR/S6 ribosomal protein axis in refractory colorectal carcinoma [30]. The limited knowledge of the widespread mechanisms and cellular targets of regorafenib prompted the oncology community to set up several clinical trials to test the efficacy of this compound in metastatic tumors such as soft tissue sarcoma (STS), renal cell carcinoma (RCC), and hepatocellular carcinoma (HCC) [31,32]. Nowadays, regorafenib has been approved by both FDA and EMA for metastatic colorectal cancer.

Lenvatinib (Lenvima 10^®^) is a broad-spectrum inhibitor that blocks the activation of several types of RTKs, including VEGFR-1 (FLT1), VEGFR-2(KDR), VEGFR-3 (FLT4), FGFR-1, FGFR-2, FGFR-3, FGFR-4, PDGFRa, RET, and c-KIT. The therapeutic indications of lenvatinib include metastatic thyroid cancer (MTC), advanced renal cell carcinoma (RCC), and hepatocellular carcinoma (HCC). Interestingly, the ability of lenvatinib to interfere with and reduce the activity of the proangiogenic VEGFs-dependent signaling pathway makes it an ideal candidate as a first line of treatment to limit the vascularization processes induced by tumor cells to support tumor growth and metastatic spread.

In differentiated thyroid cancer (DTC), bone radioiodine-resistant metastases (BM) are the main cause of low survival and greater morbidity due to the formation of several severe damages associated with skeletal-related events (SRE). Because this classical tumor antiresorptive therapy (AT) has limited benefits, an approach with concomitant therapy with multikinase TKIs and AT should be carefully considered. The validity of this strategy has been supported by a study showing that lenvatinib treatment induced a longer overall survival (OS) in DTC patients with lung metastases [33]. Future trials devoted to evaluating the outcomes and toxicity of lenvatinib in combination with AT are, therefore, warranted to determine the possible use of multikinase TKIs in metastatic DTC [34].

In the three-arm phase III trial CLEAR study, designed to compare lenvatinib plus everolimus and lenvatinib plus pembrolizumab versus sunitinib monotherapy for the treatment of RCC, lenvatinib plus pembrolizumab showed promising antitumor activity when the efficacy of this combination was assessed by progression-free survival (PFS) as primary end-point and other parameters, such as overall survival, safety, quality of life and pharmacokinetics, as secondary end-point [35]. Another phase III multi-national trial, termed REFLECT, designed to screen a possible less toxic first-line treatment of patients with unresectable HCC, demonstrated that lenvatinib, although non-inferior compared to sorafenib for overall survival, showed a better and more controllable toxicity profile [36]. Interestingly, lenvatinib is also in trial for the treatment of children and adolescents with refractory or relapsed solid malignancies, including differentiated thyroid carcinoma and osteosarcoma (NCT02432274).

Finally, one report described another approach aimed at improving the bioavailability of combined therapy of multikinase TKIs with immunochemotherapy in metastatic triple-negative breast cancer (TNBC). Using an in vivo animal model, the authors found that lenvatinib- and vadimezan-loaded synthetic high-density lipoprotein (LV-sHDL) inhibited the growth of orthotopic tumors, reduced pulmonary metastasis, and improved the survival of animals, thus underscoring the potential use of TKIs in combined therapy to treat metastatic TNBC [37].

## 3. Cyclin-Dependent Kinase Inhibitors and KRAS Inhibitors

**Figure 2:**
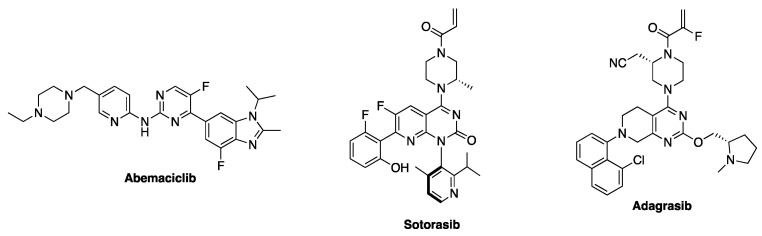
Recently FDA and EMA approved CDK inhibitors and KRAS inhibitors.

In the case of HER2 negative tumors (HER2−), the therapeutic strategies are even more challenging because this important target is missing. For estrogen receptor-positive and HER2 negative (ER+, HER2−) highly invasive breast cancer, an important new possibility to explore is represented by small molecule cyclin-dependent kinases (CDK4-6) inhibitors, such as abemaciclib, approved by FDA in September 2017 (Figure 2). Recent new promising data were obtained from “MonarchE”, a phase III randomized trial (NCT03155997) that recruited adult ER+, HER2-patients with breast cancer at high risk of recurrence from 603 sites in 38 countries. The abemaciclib treatment induced an increase in disease-free survival (DFS) and relapse-free survival (RFS) at 4 years with tolerable adverse events such as neutropenia, gastric disorders, and fatigue [38]. Abemaciclib is now in trial also for recurrent primary brain tumors (NCT032206460).

Finally, new hope for solid tumor patients comes from a compound that probably represents the first idea of a small molecule cancer cell inhibitor since it targets one of the most common, and even for years considered undruggable, mutated protein in cancers, KRAS G12C. After countless failed attempts [39], sotorasib, the first targeted drug for mutated KRAS, was eventually approved in 2021 by FDA and in 2022 by EMA for the treatment of advanced non-small cell lung cancer (NSCLC) [40]. The Overall Response Rate was 36% with a median duration of response of 10 months; diarrhea, musculoskeletal pain, hepatotoxicity, nausea, and fatigue were the most commonly reported adverse events.

Structurally targeted chemical modifications of sotorasib allowed for the synthesis of adagrasib (MRTX849), a highly selective covalent inhibitor of mutated KRAS G12C, which, in December 2022, received accelerated approval by FDA (Krazati^®^, Mirati Pharmaceutics) for the treatment of patients with KRAS G12C–mutated metastatic or unresectable NSCLC (NCT03785249 KRISTAL-1) [41]. Given these results, adagrasib was also tested in metastatic colon cancer patients showing significant therapeutic advantages (NCT03785249) [42]. The most frequent adverse events reported by patients included nausea, diarrhea, vomiting, and fatigue; nevertheless, the risk-benefit profile for the patients was considered positive by the FDA, which granted the accelerated approval. Conversely, on July 2023, EMA issued a negative opinion on the conditional marketing authorization application of Krazati because the drug did not meet the criteria for granting this type of authorization. Indeed, the pharmaceutical company could not show that Krazati fulfilled an unmet need and could not justify making the medicine immediately available to patients.

Taken together, the brilliant results achieved in KRAS G12C inhibition pave the way to new therapeutic associations between small molecules such as sotorasib and adagrasib with monoclonal antibodies targeting other pathways that currently are ruled out by the deregulated activation of mutated KRAS-linked pathways.

In Table 1 are summarized the SMIs discussed up to now (TKIs, CDK− and KRAS-inhibitors) and the clinical trials in which they have been involved over the years.

Despite the enormous efforts in the discovery of novel reliable and effective TKIs, unfortunately, there is a very arduous issue related to the intrinsic mechanism of action of this category of drugs. After some beneficial initial cycles of treatments with TKIs, a large percentage of patients do not respond anymore to the therapy because their cancer cells develop acquired drug resistance [43,44]. A point mutation within their catalytic kinase domain, decreasing the affinity of TKIs to their specific binding site by steric hindrance, accounts for the most common drug resistance mechanism. However, other concomitant additional mechanisms have been documented, such as gene amplification or overexpression, alternative splicing of RTKs, variations of elements regulating signaling pathways functionality, overexpression or mutations of drug transporters, and epigenetic modifications, including changes in microRNA formation and turnover [45].

Strategies to overcome these heavy limitations of TKIs include either experimental in vitro/in vivo and in silico approaches. The first approach relies on the development of organoids and lab-on-a-chip systems to screen the toxicity and efficacy of the new compounds. The second approach will greatly exploit the extraordinary potentiality of protein-folding computational algorithms, such as RoseTTA fold [46] and Alphafold2 [47], that allow for the identification of possible RTKs pockets domains, at a previously unattainable 3D spatial resolution, suitable to be targeted by new molecules. A better understanding of TKIs resistance is the necessary prerequisite and a potent stimulus to develop the next generations of TKIs as starting point to concretize the promises and claims of personalized medicine in cancer therapy [48].

## 4. Integrin Antagonists

Integrins are cell adhesion proteins involved in the interaction with the extracellular matrix (ECM), in the transmission of biochemical and mechanical signals between cells and their environment, and in a wide range of biological functions. In particular, αvβ3 and ανβ5 integrin subtypes turned out to be overexpressed in different cancer types, such as colon cancer, melanoma, breast cancer, and glioblastoma [49]. Therefore, in the last decades, these receptors have been widely explored as therapeutic targets in anticancer therapy [50,51,52].

**Scheme 1:**
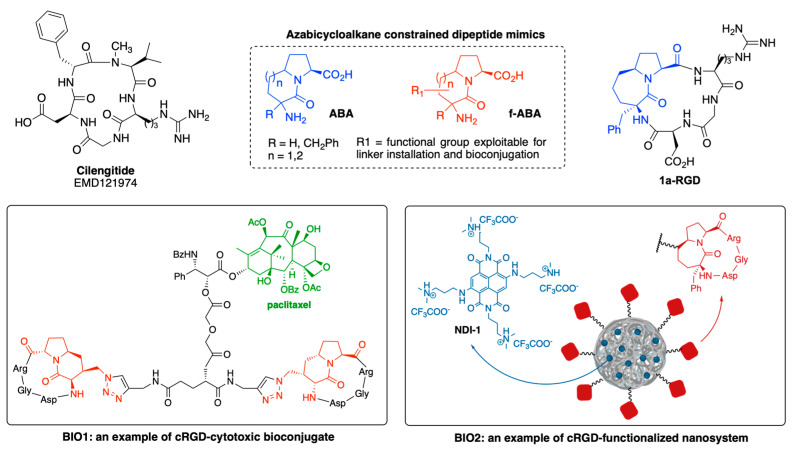
Examples of RGD-based integrin antagonists and their use in targeted drug delivery.

Since αvβ3 and αvβ5 can be expressed by both tumor cells and tumor endothelial cells, it has been speculated that drugs capable of inhibiting the adhesive function of these integrins can hamper tumor growth in at least two ways, by directly targeting the tumor and by inhibiting angiogenesis. Cyclic peptides carrying the Arg-Gly-Asp (RGD) sequence are potent antagonists of αvβ3 and ανβ5 integrin subtypes. Cilengitide (EMD121974, Scheme 1) is undoubtedly the most known integrin antagonist characterized by a cyclic peptidomimetic structure, and it has been evaluated in almost 30 different clinical trials for cancer [53]. In cilengitide, or [c(RGDfNMeV)], the presence of dipeptide d-phenylalanyl–N-methyl valine (fNMeV) allowed for fixing the RGD sequence into the correct bioactive conformation to bind in nanomolar concentrations αvβ3 and αvβ5 integrin receptors.

**Figure 3:**
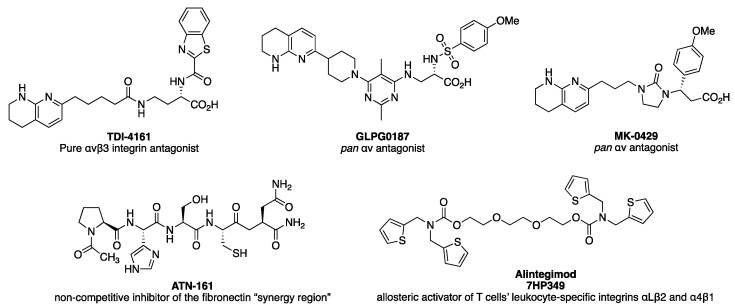
Selected integrin antagonists tested in clinical trials as anticancer agents.

Despite promising preliminary data, the use of this compound as an anticancer drug against glioblastoma has been discontinued due to failure in phase-III clinical trials, where it showed no improvement in overall patients’ survival [54]. Moreover, in a paper published in 2009, Reynolds and co-workers reported that cilengitide had the unfavorable property of paradoxically increasing angiogenesis at concentrations below its IC50 [55]. Indeed, the binding of cilengitide generates a major conformational change in αvβ3 receptor that induces it to adopt a high-affinity ligand-binding state, which is associated with the enhancement of tumor growth in vivo. To circumvent this drawback, the design of pure small-molecule antagonists such as TDI-4161 (Figure 3), which do not induce conformational changes in the receptor while maintaining high affinity and potent antitumor activity, has emerged as an area of intense interest [56]. Interestingly, the pure antagonist TDI-4161 was synthesized through rational modification of highly active αvβ3 ligand MK-0429 (vide infra) guided by a three-dimensional molecular model of the interaction between MK-0429 and the αvβ3 binding pocket, refined by molecular dynamics (MD) simulations.

The partial agonist effect of RGD-based small molecule derivatives, albeit somewhat debated, has led for some years to put aside the use of these peptidomimetic antagonists alone as anticancer agents [57]. Nevertheless, given their intrinsic very low toxicity toward healthy cells and their high affinity and selectivity for integrin overexpressed in various tumor forms, RGD-based antagonists found a second life as drug delivery systems [58,59,60].

Over the years, different groups have worked on the synthesis of cilengitide analogs by replacing the d-Phe–N-MeVal dipeptide units with various molecular scaffolds [61]. In this context, constrained dipeptide mimics, such as azabicycloalkanone amino acids (ABA, Scheme 1) [62], proved to be useful intermediates for the obtainment of cRGD antagonists such as 1a-RGD, a nanomolar inhibitor of αvβ3, and αvβ5 integrin subtypes [63]. Analogously, the synthesis of “easy-to-functionalize” azabicycloalkanone amino acids (f-ABA, Scheme 1) [64,65,66] allowed for the designing of modified cRGD derivatives such as BIO1 and BIO2, in which a side chain installed onto the azabicycloalkanone core has been exploited for the conjugation of bioactive compounds [67]. This strategy resulted in the preparation of cRGD-cytotoxic bioconjugates [68], cRGD-based imaging probes [69,70], and cRGD-functionalized nanosystems [71,72].

The high selectivity of cRGD-functionalized nanoparticles toward cancer cell lines overexpressing αvβ3 and αvβ5 integrin receptors [73] could pave the way to the use of highly active anticancer agents such as naphthalene diimide derivative NDI-1 (Scheme 1). The reverse transcriptase enzyme telomerase, overexpressed in many cancer cells, plays a key role in maintaining the telomere length for the immortal division of malignant cells. Naphthalene diimide derivatives are potent anticancer agents that act by inducing/stabilizing G-quadruplex DNA structures, thus sequestering the enzyme substrate in the single-stranded telomeric DNA [74,75,76]. As a consequence, the telomere length can gradually shorten, leading, as in the case of non-malignant cells, to senescence and apoptosis. Although some derivatives have reached clinical trials in humans [77], owing to their indiscriminate cell entry and very tight therapeutic window, naphthalene diimides can cause severe side effects, which strongly limits their applications in anticancer therapy. By loading NDI-1 into silk fibroin nanoparticles endowed with cRGD derivatives, our group observed a significantly higher cytotoxic effect on human glioma cell lines U373, which overexpress αvβ3 and αvβ5 integrin subtypes, than on D384 cell lines, characterized by a lower expression of the same integrins [78]. Moreover, the encapsulation of NDI-1 inside the nanoparticles considerably reduces its distribution in the body and, therefore, its toxicity towards healthy cells/organs.

As previously discussed in the case of cilengitide, despite the good premises of being able to synthesize novel anticancer agents by exploiting the selective binding of small molecule integrin antagonists (SMIA) to specific integrin receptors, this approach has not led to the expected clinical success. Indeed, the high adaptive capacity of cancer cells probably demands that additional biological mechanisms need to be targeted in the tumor microenvironment for SMIA to be effective.

To conclude this section, we think it could be useful to trace a brief overview of the SMIA that, in recent years, have reached clinical trials in cancer therapy [79].

The broad-spectrum (or pan) αv antagonist GLPG0187 (Figure 2) was tested in a phase Ib trial in patients with solid tumors [80]. In preclinical models and in mouse cancer models, GLPG0187 showed to be very active, inhibiting the formation and progression of bone and visceral metastases in prostate cancer and breast cancer. Although in humans turned out to be well tolerated, GLPG0187 was dismissed due to its low efficacy because the continuous infusion of this compound failed to show signs of monotherapy efficacy. The previously cited MK-0429 is a nonpeptide pan-αv integrin inhibitor that was tested in a phase I randomized double-blind study on men with hormone-refractory prostate cancer and metastatic bone disease (MDB) [81]. MK-0429 was generally well-tolerated and displayed a potential for clinical use in MDB, but, unexpectedly, an increase in serum prostate-specific antigen (SPA, a marker for disease activity) took place during the experimentation. While since 2012, no clinical activity has been reported for MK-0429 as an anticancer drug, in 2019, the interesting preclinical in vivo data on the obese ZSF1 (Zucker fatty and spontaneously hypertensive) rat model of diabetic nephropathy [82] prompted Merck to patent MK-0429 for chronic kidney disease [83].

The linear pentapeptide ATN-161 (Ac-PHSCN-NH2) is a non-competitive inhibitor of the fibronectin “synergy region” (PHSRN sequence) [84]. This region enhances the affinity and specificity of the RGD-mediated binding, thus increasing the interaction between fibronectin and the integrin receptor. ATN-161 binds to beta subunits of α5β1 and αvβ3 integrins outside the RGD-binding site, displaying antitumorigenic and antimetastatic activities in various cancers [85,86]. The peptide has been designed by introducing a cysteine residue in place of arginine of the original fibronectin PHSRN sequence, along with acetylation of the N-terminal proline and amidation of the C-terminal asparagine. The said chemical modifications were introduced to improve the pharmaceutical properties of ATN-161, which reached phase II clinical trials in patients with solid tumors such as renal cell cancer and brain and central nervous system tumors. Since 2012, no clinical activity has been reported for ATN-161 as an antineoplastic agent, but recent in vitro experiments highlighted the effectiveness of this compound in reducing the entity of SARS-CoV-2 infection [87]. These findings, which are in agreement with the hypothesis that RGD-binding integrins are co-receptors for angiotensin-converting enzyme 2 (ACE2), used by SARS-CoV-2 to entry into the host cells [88], open the door to the possible future use of integrin inhibitors in viral infections [89]. Alintegimod (7HP349) is a clinical-stage orally active immunostimulant that acts as an allosteric activator of T cells’ leukocyte-specific integrins αLβ2 and α4β1. The interaction with these receptors enhances T cell activation and adhesion, thus increasing the penetration of T cells into tumors in mouse models of melanoma and colon carcinoma [90]. Following a successful first-in-human phase I clinical trial completed at the end of 2021, Alintegimod will be entering phase Ib/IIa trials in programmed cell death 1 (PD-1) refractory solid tumors and influenza vaccination of the elderly. Moreover, in 2022 the FDA granted fast-track designation to 7HP349, in combination with a cytotoxic T-lymphocyte antigen 4 (CTLA-4) inhibitor, for the treatment of patients with unresectable or metastatic malignant melanoma [91].

In Table 2 are summarized the most interesting integrin antagonists used to treat metastatic cancers and the clinical trials in which they have been involved over the years.

## 5. Conclusions and Future Perspectives

Due to growing numbers of different classes of anticancer drugs, either in development or recently approved, the last decade has witnessed a spectacular improvement in the survival rate of patients affected by several types of solid tumors and hematologic malignancies. However, this giant leap forward has not been mirrored by a concomitant advancement of effective treatments for metastatic cancers, which accounts for more than 90% of mortality. This failure derives mainly from the still limited and vague knowledge of the many molecular mechanisms responsible for the metastatic spread that makes it really difficult and tricky to identify suitable antimetastatic targets. Undoubtedly, the use of some of the SMIs (or their derivatives) reported in this account, either alone or in combination with other therapeutic innovative drugs, could represent a cornerstone in cancer therapy.

Nevertheless, the study of metastatic processes requires an enormous joint effort by the oncology community focused on developing novel approaches and refining existing advanced technologies in cancer research. In our opinion, we do foresee that the greatest improvements will derive from the combination of two complementary and intertwined approaches: (a) new types of functional ex vivo metastatic models; and (b) single-cell spatial multiomic analyses. A recent work of Ombrato et al. [92] proposed a very elegant and clever way to detect and identify early processes in the metastatic spread of cancer cells, together with their ability to recruit and educate other non-cancer cells found in the premetastatic niche. The authors of this study engineered premetastatic cancer cells to express a releasable mCherry fluorescent protein that is then inserted into a slice of non-tumor cells. The mCherry protein is released by premetastatic tumor cells and taken up by the contiguous surrounding cells within the local tumor microenvironment (TME).

Positive mCherry TME cells can be first isolated by fluorescence-activated cell sorting (FACS) and then characterized by single-cell transcriptomics and proteomics tools to identify changes in signaling pathways induced by direct contact with metastatic cancer cells.

The exploitation of the full potential of this ex vivo model requires the implementation of reliable spatial single-cell multiomic analyses, thus allowing for the characterization at the genomic, transcriptomic, and proteomic levels of the different clusters of cells, together with their spatial localization, belonging to the metastatic niche and TME [93]. Recent improvements in the analytical procedure have clearly shown that single-cell sequencing (SCS) is a potent and affordable high-throughput tool for investigating the molecular mechanisms underlying tumor metastasis at the single-cell level [94]. Indeed, SCS can be used to study tumor heterogeneity, drug resistance, changes in the TME microenvironment, analysis of circulating tumor cells (CTCs) in liquid biopsy, and, in combination with artificial intelligence (AI), to construct metastasis-related cell maps for predicting and monitoring the dynamics of metastasis.

## Figures and Tables

**Table 1:** Summary of clinical trials involving non-approved, currently under experimentation, and marketed tyrosine kinase inhibitors, cyclin-dependent kinase inhibitors, and KRAS inhibitors.

Molecule	Target/Mechanism	Cancer Type	Clinical Trials/FDA Approval
Lapatinib	Inhibition of phosphorylation of EGFR1, HER2, HER4.	Ovarian cancer, Head and neck cancer, Prostate cancer, High-grade gliomas, Metastatic thyroid gland cancer	337 trials, 29 in progress.
		Metastatic Breast cancer	FDA approved 2010
		Advance Breast cancer	FDA approved 2006
Regorafenib	Inhibition of multiple membrane-bound and intracellular kinases.	Metastatic solid malignancies, Colon cancer, Gastro-oesophageal cancer	299 trials, 143 in progress.
		Hepatocellular carcinoma	FDA approved 2017
		Gastrointestinal stromal tumor	FDA approved 2013
		Advanced colorectal cancer	FDA approved 2012
Lenvatinib	Inhibition of the kinase activities of VEGF receptors VEGFR1, 2, 3; inhibition of FGF receptors FGFR1, 2, 3, 4.	Metastatic thyroid cancer, Hepatocellular carcinoma, Children and adolescents with refractory or relapsed solid malignancies, including differentiated thyroid carcinoma and osteosarcoma…	429 trials, 298 in progress.
		Advanced renal carcinoma	FDA approved 2021
		Advanced endometrial carcinoma	FDA approved 2021
		Unresectable hepatocellular carcinoma	FDA approved 2018
Varlitinib	Inhibition of ErbB-2 (Her-2/neu) and EGFR.	Metastatic breast cancer	12 trials (first study started in 2015), 2 in progress.
Abemaciclib	Dual inhibition of cyclin-dependent kinases 4 (CDK4) and 6 (CDK6)	Recurrent primary brain tumors, breast cancer...	199 trials, 135 in progress.
		(HR)-positive, HER2-negative advanced or metastatic breast cancer	FDA approved 2021
		Advanced or metastatic breast cancer	FDA approved 2017
Sotorasib	KRAS G12C-mutated inhibition	Non-small cell lung cancer	46 trials, 28 in progress
		KRAS G12C mutated locally advanced or metastatic non-small cell lung cancer	FDA approved 2021
Adagrasib	KRAS G12C-mutated inhibition	Gastrointestinal cancers	24 trials (first study started in 2019), 22 in progress.
		Locally advanced or metastatic KRAS G12C-mutated non-small cell lung cancer	FDA approved 2022

**Table 2:** Summary of clinical trials involving integrin antagonists.

Molecule	Target/Mechanism	Cancer Type	Clinical Trials
Cilengitide	Inhibition of phosphorylation of EGFR1, HER2, HER4.	Gliomas, lung cancer, childhood high-grade cerebellar astrocytoma, recurrent prostate cancer, glioblastoma multiforme	30 trials
GLPG0187	Non-selective αv antagonist	Solid tumors Cystic fribrosis	(phase I) (phase II)
MK-0429	Non-selective αv antagonist	Prostatic neoplasms with metastatic bone disease	(phase I)
ATN-161	α5β1 and αvβ3 integrins antagonist	Intracranial malignant glioma Renal cell cancer	(phaseI/II) (phase II)
7HP349	allosteric activator of αLβ2 and α4β1 integrins	Solid tumor	(phase I)

## Data Availability

The data presented in this study are available in this article.
